# Simultaneously Acquiring Optical and Acoustic Properties of Individual Microalgae Cells Suspended in Water

**DOI:** 10.3390/bios12030176

**Published:** 2022-03-15

**Authors:** Hongjian Wang, Ran Liao, Zhihang Xiong, Zhao Wang, Jiajin Li, Qian Zhou, Yi Tao, Hui Ma

**Affiliations:** 1 Shenzhen Key Laboratory of Marine IntelliSensing and Computation, Shenzhen International Graduate School, Tsinghua University, Shenzhen 518055, China; whj20@mails.tsinghua.edu.cn (H.W.); lijiajin21@mails.tsinghua.edu.cn (J.L.); 2 Guangdong Research Center of Polarization Imaging and Measurement Engineering Technology, Shenzhen International Graduate School, Tsinghua University, Shenzhen 518055, China; mahui@tsinghua.edu.cn; 3 Department of Photoelectric Technology, Foshan University, Guangzhou 528000, China; 2112055011@stu.fosu.edu.cn; 4 Shenzhen International Graduate School, Tsinghua University, Shenzhen 518055, China; z-wang20@mails.tsinghua.edu.cn (Z.W.); zhou.qian@sz.tsinghua.edu.cn (Q.Z.); tao.yi@sz.tsinghua.edu.cn (Y.T.)

**Keywords:** microalgae, multimodality, polarized light scattering, fluorescence, laser-induced acoustic wave

## Abstract

Microalgae play a vital role in aquatic ecological research, but the fine classification of these tiny and various microalgae cells is still challenging for the community. In this paper, we propose a multimodality technique to simultaneously acquire the polarized light scattering, fluorescence and laser-induced acoustic wave signals originated from individual microalgae cells in water. Experiments of different species of *Spirulina* and different states of *Microcystis* have been conducted to test our experiment setup, and the results demonstrate that this method can well discriminate microalgae cells with pigment or microstructural differences. Moreover, with these modalities, the consumption of absorbed energy is evaluated quantitively, and a possible way to assess photosynthesis on a single-cell level is presented. This work is expected to be a powerful technique to probe the biophysical states of microalgae in the aquatic ecosystem.

## 1. Introduction

Microalgae play a vital role in aquatic systems [1]. Some microalgae species are likely to cause harmful blooms, and some species are the important food source for marine organisms and raw materials for human health products [2,3]. In situ monitoring the categories and growing states of microalgae is important to forecast the changes of the aquatic environment and then take timely measures [4]. However, microalgae have a wide variety of pigment, size, shape and microstructure, which causes the difficulties in discriminating them [5].

Recently, lots of imaging technologies have been developed to in situ classify and monitor the state of the microalgae, such as holographic microscopy [6], Imaging FlowCytobot [7] and dark-field imaging microscope [8]. However, the technologies based on images are easily time-consuming, and their quality and efficiency are easily subjected to the tradeoff between resolution and visual field. To observe the microstructure clearly, scanning electron microscope and transmission electron microscope are two popular tools [9], but the sample preparation and experimental operations are complicated. To probe the suspended microalgae cells, spectrophotometry has been reported to evaluate the microalgae biomass with the relationship between the absorbance and cell density [10]. Also, the high-frequency echosounder can be applied to investigate the dynamics of the gas-bearing cyanobacterial *Microcystis*, by taking advantages of the strong backscatterer’s properties [11]. These methods measure the biomass of microalgae cells in bulk volume, which leads to the low resolution and limits their further applications in further classification. On the contrary, the individual measurement separates the individual particle from the ambient and then gets its information of different modalities, which effectively enhances the classification ability. Recently, the flow cytometer, as a kind of individual measurement, can be compactly built with the integrated microfluidic chips and optical components [12]. However, large particles easily block the flow system, and the pretreatments are required before the measurement. Today, the fast and effective tools for the microalgal classification are still challenging for the community.

During the interaction between light and microalgae, scattering and absorption are two basic processes [13]. Scattering of light is usually related to the physical information of the particles, and the polarization state of light will change after the scattering happens [14]. Polarized light-scattering measurement, as an emerging technique, has been applied to characterize muscle tissue [15] and aerosol particles [16]. Also, polarized light scattering has been reported to characterize the structures of microalgae, and the polarization parameters are useful in classifying different categories of microalgae [17,18].

Microalgae usually have intracellular chromophores, such as chlorophyll and carotenoids [19]. After the chromophores absorb light, the optical energy can be consumed mainly in three aspects, which are photosynthesis, fluorescence, and heat [20]. In the past decades, fluorescence measurement has been popular to be applied in probing the aquatic environment. Since the intensity of fluorescence is closely related to the composition and contents of the intracellular pigments, the measurement of autofluorescence can be applied to differentiate some categories of microalgae [21].

After the high-power laser irradiates the absorption material, the thermal effect is likely to cause mechanical vibration of the surrounding medium, which can be detected as the laser-induced acoustic wave [22]. Laser-induced acoustic wave effects, together with ultrasound detection, have been developed and applied in biomedical and diagnostic fields [23,24]. With the labeling of the contrast agents, some literature studies report applying laser-induced acoustic measurement to assess the circulating metastatic melanoma cells in blood [25,26]. Also, the fluorescence life and photoacoustic signals of unicellular diatom algae can be useful in reconstructing the images for diatom cells [27]. However, due to the low light absorption of the individual microalgae cells, the simultaneous and in situ measurement of their label-free autofluorescence and laser-induced acoustic signals are still difficult and unreported [28]. Moreover, the measurements based on the optical modality may meet their bottlenecks in fine differentiation of some microalgae. Therefore, the multimodality methods are always used to obtain the microalgal information of multiple aspects, while classifying the diverse microalgae.

In this work, a multimodality method is presented to simultaneously acquire the polarized light scattering, fluorescence and acoustic signals, and all of them are originated from the individual microalgae in water. Subsequently, we designed three groups of experiments to test our experiment setup. These experiments were designed aiming to cover three typical challenging groups, such as the differentiation of different categories of microalgae, the fine classification of different species of *Spirulina* and the identification of different states of *Microcystis aeruginosa* under sonication treatment. Experiments were conducted and the results demonstrate that this method can differentiate unicellular flagellate microalgae, *Euglena* and *Cryptophyta*, and this method can well characterize and differentiate two common species of *Spirulina* and different states of *Microcystis aeruginosa*.

The photosynthetic energy storage efficiency is one of the important indicators to analyze the status of the microalgae, and it is related to the active biomass of phytoplankton assemblages [29]. However, the detailed photosynthetic energy storage efficiency for each composition in aquatic suspension is difficult to retrieve. In this work, we give the prospect to quantitatively evaluate the consumption of the absorbed energy and assess the photosynthetic energy on a single-cell level, which may provide the possible way to study the photosynthesis of the individual microalgae. Subsequently, the measured scattering signal is further discussed in detail.

## 2. Methods

### 2.1. Samples

*Euglena* is a unicellular flagellate microalgal species, which moves fast and is distributed widely in freshwater, it is an important food source of some marine organisms, and it can purify polluted water [30]. *Euglena* has several chloroplasts and contains several kinds of pigments like chlorophyll-a (Chl-a) and carotenoids [31]. The sample of *Euglena* is supplied by Freshwater Algae Culture Collection at the Institute of Hydrobiology, Chinese Academy of Sciences. *Cryptophyta* is also a common category of unicellular flagellate microalgae, is considered as one of the important taxa of photosynthetic microorganisms, and its shape is flat and asymmetric. The pigments of *Cryptophyta* include Chl-a and phycobilin, and the phycobilin is rare, which can be considered as the feature pigment of *Cryptophyta* [32]. The sample of *Cryptophyta* is supplied by Shanghai Guangyu Biological Technology Co., Ltd.

*Spirulina* is a kind of economic blue-green algae growing in warm alkaline environments, which is the rich source of pigments. In *Spirulina* category, *platensis* and *maxima* are two well-known and important species, and lots of comparison studies were conducted between them [33,34]. However, the taxonomies of different species of *Spirulina* are still challenging, even though they have some differences in their growing conditions and adaptability [35]. These two species of *Spirulina* are provided by Freshwater Algae Culture Collection at the Institute of Hydrobiology, Chinese Academy of Sciences.

Cyanobacterial bloom is a global aquatic issue, and the bloom is toxic and greatly threatens the aquatic environment and human beings [36]. The *Microcystis* category is the dominant species during the cyanobacterial blooms. To deal with the blooms, sonication treatment (ST) has been reported as a popular non-polluting method [37]. However, the tools to monitor the change of states of *Microcystis* are still rarely reported. The field sample of *Microcystis* was collected during the bloom in a fish pond of Zhuhai city (22°8′ N, 113°16′ E), on 26 August 2021.

For the measurement of each sample, 100 μL microalgae cells are firstly sampled from the original concentrate and added into the sample pool of distilled water. Then, these categories of microalgae cells are measured by the experiment setup, respectively. After that, different modalities of the sample are analyzed and compared with others. Since the proposed measurement technique is designed for probing microalgae individually, the concentration of sampled microalgae cells does not matter as long as the concentration is less than $10^{5}$ cells per millimeter (mL). Note that the microalgae in these experiments are sampled in their exponential growth states, and the statistical analysis in this work is based on the probability distribution of more than 1000 records.

### 2.2. Experiment Setup

**Figure 1:**
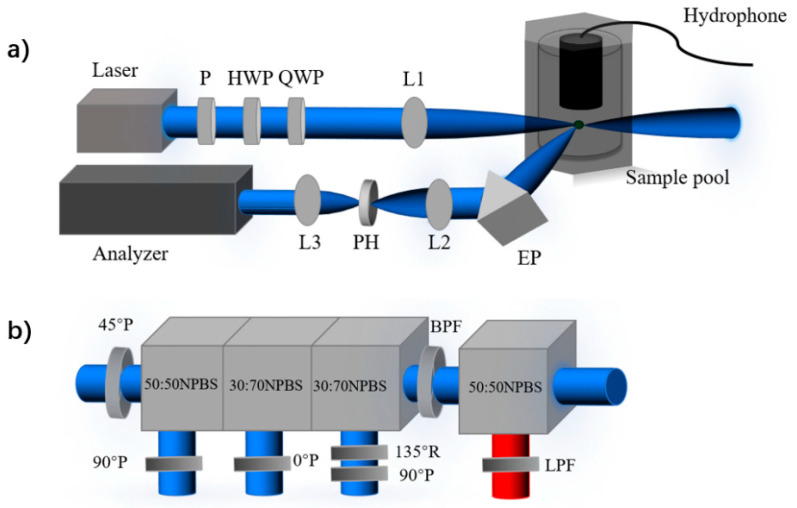
(**a**) The schematic diagram of the experiment setup. Linear polarizer (P); half-wave plate (HWP); quarter-wave plate (QWP); convex lenses (L1, L2, and L3); equilateral prism (EP); pinhole (PH). (**b**) Analyzer of the polarization and fluorescence. Non-polarizing beam splitter cube (NPBS); long-pass filter (LPF); bandpass filter (BPF); quarter-wave plate (R); polarizer (P).

The experiment setup, as shown in Figure 1, is built to measure the polarized light scattering, fluorescence and laser-induced acoustic signals originated from the individual microalgae in aquatic suspensions. The experiment setup uses a laser with the 445 nm wavelength, the maximum power is 1.0 W, and the 445 nm wavelength ensures relatively large absorption of intracellular pigments of most microalgae. The light firstly passes through the polarizer (P) to get the linear polarization state of light, then the polarization state is modulated by the use of a rotating a half-wave plate (HWP) and a rotating quarter-wave plate (QWP). After the polarization state of light is modulated, the light beam is focused on the scattering volume with the lens (L1). In the sample pool, a magnetic stirrer at the bottom of the sample pool rotates at the speed of 100 rounds per minute to keep the particles suspended. The stirrer is oblate spheroid to reduce the sound originated from the stirring process, which lowers the background of the acoustic detection.

**Figure 2:**
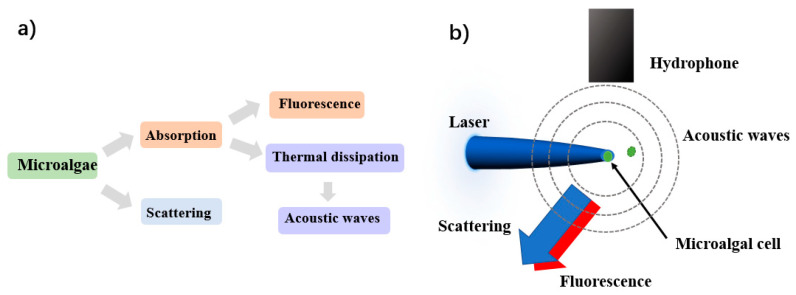
(**a**) The interaction processes between light and microalgae cell; (**b**) Detection schematic of the measurement.

Note that the power of the incident light is 0.5 W during the measurement, and the light is focused into the scattering volume about 0.01 μL. Once microalgae pass through the scattering volume, scattering and absorption of light happen, the interaction processes are shown as Figure 2. As illustrated in Figure 2a, some of the absorbed light energy is used in the radiative relaxation to emit fluorescence, and some is consumed in the non-radiative thermal dissipation, which heats up the surrounding and causes the vibration of the medium [24]. The mechanical vibration can be detected as acoustic wave. In our work, the acoustic waves are considered as a series of spherical waves, and they are detected by the hydrophone (HP-01-020S), which is located about 2 cm above the center of the scattering volume, as shown in Figure 2b. The sensitivity of this hydrophone is −185 dB re 1 V/µPa, and the bandwidth range is from 20 Hz to 20 kHz. Due to its small size, the hydrophone with 15 mm diameter and 32 mm length can be fixed and held in the sample pool. For the optical signals, including fluorescence and scattered light, they are simultaneously received at the scattering angle of backward 120°. The polarized light scattering at backward 120° has previously been proven to be sensitive to microstructures of the samples [17]. Different from the scattering, the fluorescence expands uniformly to the surrounding [30]. By adjusting the aperture and focal length, the numerical aperture of the receiving optical system is 0.087, then the half angle of the field of view is 7.5°. Then, the solid angle Ω can be calculated as 2π[1 – cos (7.5°/2)], which is 1.35 milli-steradian. Since the steradian of the whole sphere is 4π, the collected fluorescent energy takes 0.0011 of the whole.

To analyze the optical signals, the received light firstly passes through an equilateral prism (EP, #47-280, Edmund, made in Singapore), then light is focused by lens (L2) and passes through the pinhole (PH, #84-068, Edmund, made in Tokyo, Japan), to block the stray light. Subsequently, the light gets collimated through lens (L3), into the analyzer of fluorescence and scattered light. By limiting the focal spot size and the size of PH, the scattering volume can be confined to less than 0.01 μL, and individual particles detection can be realized when the concentration of the suspended particles is lower than $10^{5}$ particles per milliliter (mL).

Inside the analyzer, the light is firstly split into two light beams by the non-polarizing beam splitter cube (BS004, Thorlabs, made in Newton, NJ, USA), and the fluorescence can be separated with the long-pass optical filter of 460 nm. The intensity of light is integrated from the wavelength 460 nm to 950 nm, which is stored as *F*. Then the residual light passes a bandpass optical filter with the wavelength 445 ± 10 nm, and enters the polarization state analyzer. To analyze the polarization state of the scattering light, the scattering light is separated into 4 parts with three non-polarization beam splitter cubes and polarizers to obtain three linear polarization components and one right circular polarization [23].

**Figure 3:**
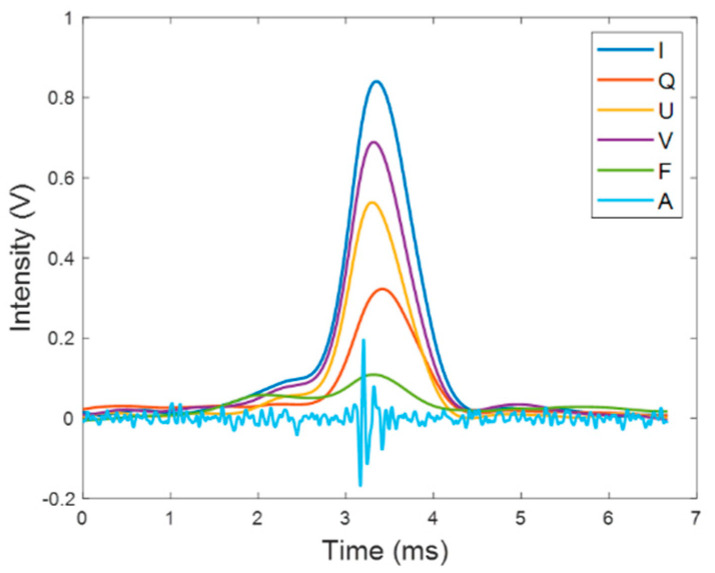
Simultaneously recorded temporal optical signals (*I*, *Q*, *U*, *V*, *F*) and acoustic signal (marked as *A*).

These polarization components are transformed into four analog voltages by the photomultiplier (HKPMD300kDTM, Suzhou Biaozhang Electronic Technology, made in Suzhou, China), and calibrated to obtain the Stokes vector, S. The Stokes vector S of individual suspended cells can be represented as ${\left[I,Q,U,V\right]}^{T}$. After measuring for some time, a series of temporal pulses can be recorded in the data acquisition card (FCFR-USB2066, Beijing Fcctec Technology, made in Beijing, China). One of the recorded signals is shown in Figure 3; it can be seen that all the pulses are detected simultaneously, and these overlapped signals correspond to an individual microalgae cell. The intensity of the signal is stored in the data acquisition card whose detecting voltage is ranging from −5 V to 5 V.

In Figure 3, it can be found that the duration time of optical pulses is about 1 ms, while the duration time of the acoustic signal is much shorter compared with the optical signals. The acoustic signal travels at the speed of 1433 m/s in distilled water, the distance between the prober end and the center of scattering volume is approximately 2 cm, so the sound will travel to the prober end in $1.4\times 10^{-5}$ s. Considering that the sampling frequency of the acquisition card is 300 kHz, this means that there is only approximately a four-sampling-point delay, which is short enough to be ignored in this work.

### 2.3. Optical and Acoustic Signal Analysis

The acoustic signal is triggered by a single sound source, and the frequency of the acoustic signal is related to the features of heat dissipation during the interaction between the light and microalgae. If the microalgae cell absorbs more light, the thermal effect is much stronger, which may have a higher frequency [22]. Herein, fast Fourier transform (FFT) is used to calculate the frequency of the acoustic signal, and obtain the transformed frequency spectra $\left|P_{1}\left(f\right)\right|$. In the frequency spectra of the acoustic signal, the frequency $f_{max}$ at the maximum amplitude in the spectra is recorded as the feature parameter of the acoustic signal, and it is used to discriminate different categories of microalgae hereafter.

The optical signals are recorded simultaneously, including four channels of polarized light scattering and one channel of fluorescence. The whole optical pulses shown in Figure 3 are averaged into one number, that is, $I_{m},\;Q_{m},\;U_{m},\;V_{m},F_{m}$. Three polarization parameters, *q*, *u*, and *v*, are defined by Equation (1), and they are ranging from −1 to 1.

(1) $$q=\frac{Q_{m}}{I_{m}},\;u=\frac{U_{m}}{I_{m}},\;v=\frac{V_{m}}{I_{m}}$$

To make the best use of q, u, and v, the linear discrimination analysis (LDA) is applied to find a projection axis, and weight these three polarized light parameters into one projected value f(q,u,v). The training target is to maximize the between-class difference ${\left|\mu_{1}-\mu_{2}\right|}^{2}$ and minimize the within-class variability $\left(\delta_{1}^{2}+\delta_{2}^{2}\right)$ in the projected space, the optimization target L can be formulated as Equation (2),

(2) $$L=\frac{{\left|\mu_{1}-\mu_{2}\right|}^{2}}{\left(\delta_{1}^{2}+\delta_{2}^{2}\right)}$$

To describe the relationships of all these measured parameters, the variance inflation factor (VIF) is used to evaluate the multilinearity of every parameter to others. The value of VIF is defined as Equation (3),

(3) $$VIF=\frac{1}{1-R_{i}^{2}}$$

where $R_{i}$ is the correlation coefficient when regressing the *i_th_* feature parameter on the remaining feature parameters. Generally, if the value of VIF of the feature parameter is larger than 10, we can consider this feature parameter is collinear with the other variables, otherwise, this feature parameter is independent to the others.

## 3. Results

### 3.1. Discriminations between Cryptophyta and Euglena

**Figure 4:**
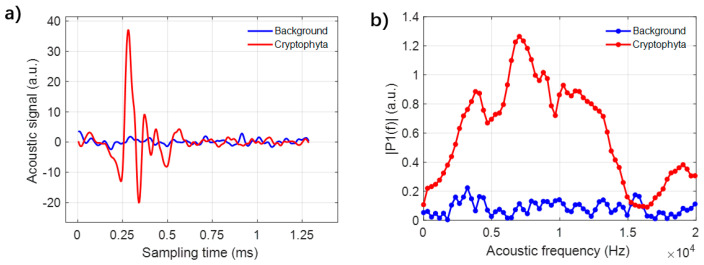
(**a**) The acoustic signals of *Cryptophyta* and the background; (**b**) The fast Fourier transform (FFT) spectra of *Cryptophyta* and the background signal.

Firstly, we take *Cryptophyta* for example to introduce the analysis of the acoustic signal. When an individual cell of *Cryptophyta* passes through the scattering volume, the laser-induced acoustic wave is recorded in our data acquisition card. The recorded temporal acoustic signal is shown in Figure 4a, as the red line. Compared with the background noise, the acoustic signal has an obvious vibrating fluctuation. Then, the frequency spectra of the temporal acoustic signal are calculated by FFT, as shown in Figure 4b. For the frequency spectra, the values of the frequency of the background noise fluctuate below 0.2, while the values of the frequency spectra of *Cryptophyta* are significantly larger than those of the background noise. We can find that the maximum amplitude is located at the frequency of about 7000 Hz, which is extracted as the feature acoustic frequency and applied for further analysis. Note that the maximum feature acoustic frequency is named as acoustic frequency hereafter.

**Figure 5:**
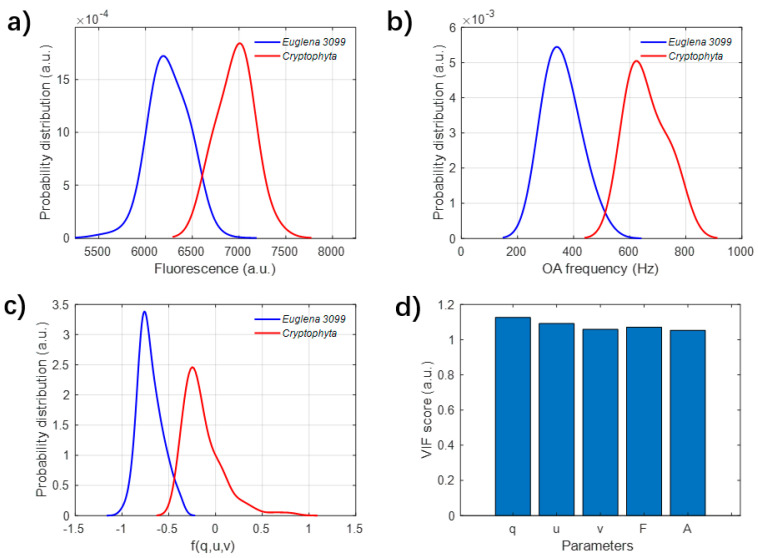
(**a**–**c**) The probability distributions of (**a**) acoustic frequency, (**b**) the fluorescence and (**c**) the projected polarization parameter f(q,u,v) of *Euglena* and *Cryptophyta*; (**d**) the variance inflation factor (VIF) scores of these feature parameters.

The signals of *Euglena* and *Cryptophyta* samples are measured and compared with each other. After measuring the suspended samples for about ten minutes, a series of the acoustic and optical signals are simultaneously recorded and respectively analyzed. The acoustic and optical features are analyzed and used to characterize the samples, including the acoustic frequency, fluorescence and integrated polarization parameters. Then, their probability distributions of these two categories were counted and are shown in Figure 5.

The distributions of acoustic frequency in Figure 5a indicate that the acoustic frequency of *Cryptophyta* is mainly located around 7000 Hz, while the frequency of *Euglena* is mostly located around 6000 Hz. For the optical parameters, both the distributions of fluorescence F in Figure 5b and the projected polarization parameter in Figure 5c can also well discriminate these two categories. In short, all of the acoustic and optical parameters can differentiate these two categories of unicellular flagellate microalgae. To describe the relationships of all these measured parameters, the evaluation of VIF is applied to evaluate the multicollinearity, and the calculation background is explained in Section 2.3. From Figure 5d, all VIFs of the parameters, including q, u, v, F and the acoustic frequency (A), are much less than 10. According to the meaning of VIF defined in Equation (3), these results in Figure 5d indicate that all of the measured parameters are independent from each other, and all of these parameters contain uncorrelated and different information of particles.

### 3.2. Fine Classification between Two Species of Spirulina

**Figure 6:**
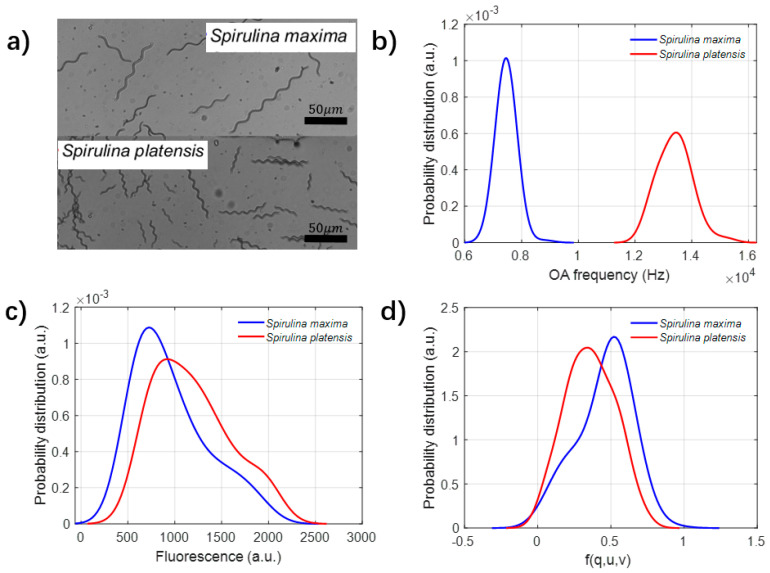
(**a**) The microscopic photo of *Spirulina* platensis and *Spirulina* maxima. (**b**–**d**) The probability distribution of acoustic frequency, fluorescence and f(q,u,v).

The fine classification between different species of *Spirulina* is challenging. The common method to discriminate *Spirulina* platensis and *Spirulina* maxima is by comparing and analyzing the microstructural differences, such as helicity and trichome size. However, it is difficult to classify them by original imaging methods with a low resolution; the microscopic pictures can be referred to Figure 6a.

The distributions of optical and acoustic parameters are measured and analyzed, and the results are shown as Figure 6b–d. Figure 6b shows that the acoustic frequency can effectively differentiate between these two species of *Spirulina*, while the frequency of *Spirulina* platensis is much larger than that of *Spirulina* maxima. However, both the fluorescence and scattering meet their bottleneck in the differentiation between these two species. The fluorescence intensity of *Spirulina* platensis is slightly larger than that of *Spirulina* maxima, but most of them are overlapped, as shown in Figure 6c. These results imply that the acoustic frequency is potentially a powerful indicator to discriminate these different samples. Both the fluorescence and acoustic frequency are physically originated from the pigment absorption; it can be inferred that the acoustic frequency may be sensitive and enlarge the absorption differences.

### 3.3. Classification among Two States of Microcystis

**Figure 7:**
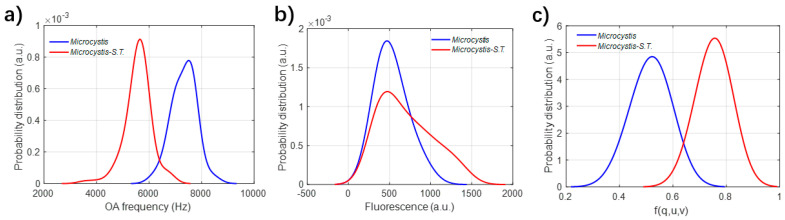
(**a**–**c**) Statistical probability distribution of different parameters. (**a**) Frequency of optoacoustic signal; (**b**) Fluorescence; (**c**) Projected polarization parameter f(q,u,v).

The mechanism of sonication treatment (ST) is to destroy the intracellular gas vesicle, to adjust the floating–sinking behavior of the harmful algae with intracellular vesicle. The samples of *Microcystis aeruginosa* of the controlled group and after ST are measured, respectively, by the experimental setup. The statistical distributions of different parameters are shown in Figure 7. It can be seen that both the acoustic frequency and intensity of fluorescence can be applied to classify these two categories. The fluorescence intensity of some algal cells after ST is higher than the controlled group, but there are a lot of overlaps between them, which disables the discrimination of these states.

**Figure 8:**
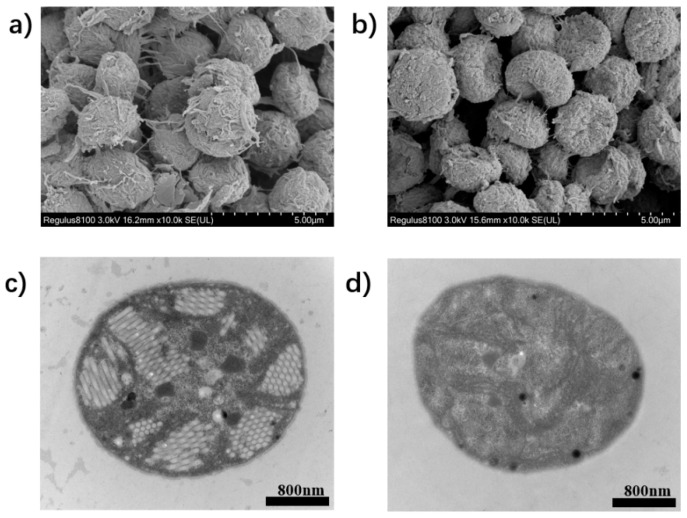
(**a**–**d**) SEM pictures of *Microcystis aeruginosa* cell (**a**) before and (**b**) after ST; TEM pictures of *Microcystis aeruginosa* cell (**c**) before and (**d**) after ST.

After ST, the pictures are taken using the scanning electron microscope (SEM) and transmission electron microscope (TEM), to observe the changes inside and outside the cell, as shown in Figure 8. From the SEM pictures shown in Figure 8a,b, the cells after ST are collapsed, and the adhesion proteins between the cells are mostly broken. Also, the intracellular gas vesicles are destroyed entirely after ST from the photos of TEM, as shown in Figure 8c,d, while the cytoplasm and membrane remain mostly intact. In short, the polarization measurement can be used to characterize the microstructural changes, while the acoustic frequency is potentially a sensitive indicator to characterize the change of the absorption behavior.

## 4. Discussions

The absorbed light is mainly consumed in the excitation of fluorescence and heat dissipation, and they are recorded as fluorescence and acoustic signal in our work, respectively, then these parameters can be evaluated in the energy aspect. For fluorescence, the original fluorescent voltage F(t) is stored in the data acquisition card, the solid angle Ω of the receiving light accounts for 0.0011 of the whole, and the sensitivity of photomultiplier is 0.2 nW/V. Considering that the light beam is equally spitted into two beams with the splitter, the total energy of fluorescence $E_{F}$ of an individual particle can be calculated by Equation (4), and the recording time is started from the initial time $t_{0}$ to the end time $t_{1}$,

(4) $$E_{F}=\frac{2\times 0.2}{\Omega }\times \overset{t_{1}}{\underset{t_{0}}{\int }}F\left(t\right)dt$$

For the simultaneously recorded acoustic energy, the sensitivity of the hydrophone is 2500 Pa per voltage, the temporal change of pressure p(t) can be derived. The sound propagation process can be treated as a dipole radiation model [38], and the acoustic energy $E_{s}$ can be calculated by Equation (5), from the initial time $t_{2}$ to the end time $t_{3}$,

(5) $$E_{s}=\frac{2\pi R^{2}}{3\rho c}\overset{t_{3}}{\underset{t_{2}}{\int }}p^{2}\left(t\right)dt$$

where R = 2 cm is the distance from the center of scattering volume to the probe end of the hydrophone, c=1433 m/s is the speed of sound when propagating in water and p(t) is the axial acoustic pressure of the signal at the end of hydrophone. ρ = 1000 kg/m^3^ is the density of the surrounding distilled water.

By integrating the temporal intensity with Equations (4) and (5), the energy can be quantitively evaluated for each cell. Then the statistical analysis is conducted for all these six samples, their averaged energy and standard deviation are shown in Table 1. Considering the features of the statistical distribution, the mean value of the energy can reflect the overall microalgal features. For the mean values in Table 1, it can be seen that the fluorescent energy of *Euglena* is lower than *Cryptophyta*, but the acoustic energy is higher than *Cryptophyta*. To compare the consumption of different forms of energies, $R_{e}$ is defined as the ratio of the averaged fluorescent energy $E_{F}$ to the acoustic energy $E_{s}$, to quantitively evaluate the utilization of the absorbed energy. The ratios $R_{e}$ of *Euglena* and *Cryptophyta* are 1.33 and 4.36, respectively. In group two, a huge difference can be found between these two species of *Spirulina*. Both the fluorescent and acoustic energies of maxima are much lower compared with the platensis, and form the value of $R_{e}$ the fluorescent energy of maxima is 25.68 times larger than the acoustic energy, while the fluorescent energy of platensis is only 0.63 of the acoustic energy. Moreover, comparing different states of Microcystis sample in group 3, after ST, the fluorescent energy increases while the acoustic energy decreases, and the ratio $R_{e}$ changes from 0.24 to 1.62.

From the results in Table 1, for *Euglena*, the standard deviation of the fluorescent energy is a little big, while the standard deviation of acoustic energy is small. The standard deviation of *Cryptophyta* sample is small both in fluorescent and acoustic energies. Since they are unicellular, these standard deviations may result from different routes when the microalgae cells pass through the scattering volume. Also, different states of microalgae cells will influence the absorption of light [39]. Compared with *Euglena* and *Cryptophyta*, the groups of *Spirulina* and Microcystis have big standard deviations in the fluorescent and acoustic energies. Apart from the standard deviation caused by the movement route of the microalgae cells, by the microscopic images of *Spirulina* and Microcystis shown in Figure 6 and Figure 8, different growth states of *Spirulina* and gathering states of Microcystis will significantly bring the deviations.

Photosynthesis consumption is one of the destinations of the absorbed energy, besides the fluorescent excitation and the thermal dissipation. The photosynthesis of an individual microalgae can be further assessed together with the pre-measured intrinsic parameters. Referred to the absorption spectrum of single isolated *Euglena* cell, which has been measured and reported previously [40], the absorbance Abs at the 445 nm is about 0.7. For the light-response curves of electron transport rate of the *Euglena* [41], the saturated irradiance $I_{0}$ is about 2000 $\mu \mathrm{mol}\cdot m^{-2}\cdot s^{-1}$, which is equally 215 nW in our case. By Beer–Lambert’s law, the intensity of the transmission light $I_{1}$ can be calculated by Equation (6),

(6) $$I_{1}=\frac{I_{0}}{10^{Abs}}$$

The intensity of the absorbed light $I_{a}$ is $I_{0}-I_{1}$, which is about 172 nW. After the algal cell moves into the scattering volume, the scattering intensity increases smoothly, and the cell begins to absorb light. Once the cell absorbs saturated light energy, the acoustic signal is detected. The time interval between these two events is the duration time of the absorption process, which is about 0.4 ms in our case, then the total absorbed light energy is estimated to 68.8 pJ. Compared with Table 1, the estimated overall fluorescent and acoustic energy is 60.95 pJ, accounting for 88.59% of the absorbed energy, and the left 11.41% is likely to be consumed in the photosynthetic process. Since the transmission distances between the microalgae cell (in the scattering volume) and the optical or acoustic detectors are quite small, here we omit the transmission attenuation of optical or acoustic energy in water. Even so, the photosynthesis consumption can be evaluated quantitively.

By dividing the accumulating time of energy, the power can be further evaluated and compared. Here, the power is analyzed for the received light around backward 120°. For *Euglena*, the received energies of scattering and fluorescence are 25.91 nW and 7.90 nW, and the power of the measured acoustic signal is about 52.38 nW. Above all, the simultaneous measurement of these parameters requires sensitive detectors, and the tests should be conducted in a proper environment to improve the signal-to-noise ratio.

Moreover, the measured signal is further discussed. When the particles pass through the scattering volume, the scattering intensity rises and falls smoothly like those pulses in Figure 3, and the pulse duration is around 1 millisecond. In addition, our analysis focuses on the measured signals similar with those in Figure 3. Unexpectedly, during our experiments, some narrow peaks are accompanied with the measured scattered light signals. Additionally, the fluorescence and acoustic signals can be recorded simultaneously together with these narrow peaks. Since the duration time of these narrow peak is very short (less than 20/mu s), these peaks may not be contributed by particles. Instead, it may be originated from the absorption matter on the molecular level. Due to the short duration time, these peaks contribute very little energy to the total energy (less than 1%). In this work, the narrow peaks are removed before the statistical analysis by the low-pass filtering and averaging filtering, since our targeted microalgae cells are micron scale.

In this paper, we first time propose a well-designed multimodality method to simultaneously acquire the optical signals, including polarized light scattering and fluorescence, and laser-induced acoustic signals of the individual microalgal cells suspended in water. Due to the simultaneous measurement, this method gives more information about the diverse microalgae than the combination of the separate modalities. Additionally, since both optical and the acoustic signals are so transient and weak that they are difficult to detect at the same time, we have never seen any similar method reported for the individual microalgae suspended in water.

Even though methods of each modality have been presented separately in literatures, in our work they are deliberately selected to cover the main consumptions of the optical energy interacted (i.e., both scattered and absorbed) instantaneously with the individual microalgal cells. Based on the simultaneous measurement and the multimodal-energy acquisition, we further show a way to evaluate the photosynthesis on the single-cell level.

For the future application, microalgae cells are generally distributed in complex aquatic environment, and the microalgae cells are inevitably mixed with organic matter, contaminants and microorganisms. Due to the advantages of the measurement of individual particles, the targeted microalgal cell can be firstly separated from all the other particles when it is measured. For the dissolved matter or particulate matter less than submicron in the scattering volume, they will contribute to the background, but the particles such as the microalgae will generate the signal of temporal pulses. For the experiments in this work, there are some dissolved matter or tiny debris from the cultured microalgal suspensions, but still our method can successfully get the signals of the microalgal cell. In the current configuration of the setup, the high particulate concentration may challenge the measurement of individual particles and then disable the method. However, in these cases, the method can be further improved, such as shortening the optical length in water or sampling the water in a transparent tube, etc.

## 5. Conclusions

In this work, a method is presented to simultaneously acquire the optical and acoustic properties of individual microalgae suspended in water. The conceptual experiment setup is designed to simultaneously measure the polarized light scattering, fluorescence and light-induced acoustic signals of individual microalgae. With the experiments results of different categories of microalgae, we demonstrate the power of our proposed method and find that the applicability of these modalities is quite different. The two unicellular flagellate microalgae, *Euglena* and *Cryptophyta*, are well differentiated by each of these three modalities. However, only the acoustic frequency is capable of classifying different species of *Spirulina.* Also, the acoustic frequency and the polarization parameters can both differentiate two states of *Microcystis aeruginosa*. Moreover, with all the simultaneously acquired modalities, the method gives the prospect to quantitatively evaluate the consumption of the absorbed energy, which is a possible way to assess the photosynthesis on a single-cell level. Such that, this multimodality method promises the powerful ability to retrieve the multiple-aspect properties of the microalgae. The future instruments based on this concept may potentially be a powerful tool to monitor the microalgae in the aquatic ecosystem.

## Figures and Tables

**Table 1:** Quantitative analysis of acoustic and optical energy.

	Group 1		Group 2		Group 3	
	*Euglena*	*Cryptophyta*	*Spirulina* *platensis*	*Spirulina* *maxima*	*Microcystis*	*Microcystis* (S.T.)
$E_{F}\;\left(\mathrm{pJ}\right)$	34.76 ± 25.59	50.14 ± 5.59	3318.90 ± 415.22	454.80 ± 137.09	271.30 ± 118.10	479.44 ± 147.36
$E_{s}\;\left(\mathrm{pJ}\right)$	26.19 ± 1.56	11.51 ± 1.23	5308.18 ± 106.23	17.71 ± 5.71	1116.67 ± 838.85	296.72 ± 277.51
$R_{e}\;\left(a.u.\right)$	1.33	4.36	0.63	25.68	0.24	1.62

## Data Availability

Data sharing not applicable.
